# Limited Impact of SARS-CoV-2 on the Human Naso-Oropharyngeal Microbiota in Hospitalized Patients

**DOI:** 10.1128/spectrum.02196-22

**Published:** 2022-11-09

**Authors:** Christopher K. C. Lai, Man Kit Cheung, Grace C. Y. Lui, Lowell Ling, Jason Y. K. Chan, Rita W. Y. Ng, Hiu Ching Chan, Apple C. M. Yeung, Wendy C. S. Ho, Siaw Shi Boon, Paul K. S. Chan, Zigui Chen

**Affiliations:** a Department of Microbiology, Faculty of Medicine, The Chinese University of Hong Kong, Hong Kong SAR, China; b Department of Medicine and Therapeutics, Faculty of Medicine, The Chinese University of Hong Kong, Hong Kong SAR, China; c Department of Anaesthesia and Intensive Care, Faculty of Medicine, The Chinese University of Hong Kong, Hong Kong SAR, China; d Department of Otorhinolaryngology, Head and Neck Surgery, Faculty of Medicine, The Chinese University of Hong Kong, Hong Kong, SAR China; e Stanley Ho Centre for Emerging Infectious Diseases, Faculty of Medicine, The Chinese University of Hong Kong, Hong Kong SAR, China; University of Georgia

**Keywords:** COVID-19, SARS-CoV-2, 16S rRNA, naso-oropharyngeal microbiome, hospitalized

## Abstract

Numerous studies have reported dysbiosis in the naso- and/or oro-pharyngeal microbiota of COVID-19 patients compared with healthy individuals; however, only a few small-scale studies have also included a disease control group. In this study, we characterized and compared the bacterial communities of pooled nasopharyngeal and throat swabs from hospitalized COVID-19 patients (*n* = 76), hospitalized non-COVID-19 patients with respiratory symptoms or related illnesses (*n* = 69), and local community controls (*n* = 76) using 16S rRNA gene V3-V4 amplicon sequencing. None of the subjects received antimicrobial therapy within 2 weeks prior to sample collection. Both COVID-19 and non-COVID-19 hospitalized patients differed in the composition, alpha and beta diversity, and metabolic potential of the naso-oropharyngeal microbiota compared with local controls. However, the microbial communities in the two hospitalized patient groups did not differ significantly from each other. Differential abundance analysis revealed the enrichment of nine bacterial genera in the COVID-19 patients compared with local controls; however, six of them were also enriched in the non-COVID-19 patients. Bacterial genera uniquely enriched in the COVID-19 patients included *Alloprevotella* and *Solobacterium*. In contrast, *Mogibacterium* and *Lactococcus* were dramatically decreased in COVID-19 patients only. Association analysis revealed that *Alloprevotella* in COVID-19 patients was positively correlated with the level of the inflammation biomarker C-reactive protein. Our findings reveal a limited impact of SARS-CoV-2 on the naso-oropharyngeal microbiota in hospitalized patients and suggest that *Alloprevotella* and *Solobacterium* are more specific biomarkers for COVID-19 detection.

**IMPORTANCE** Our results showed that while both COVID-19 and non-COVID-19 hospitalized patients differed in the composition, alpha and beta diversity, and metabolic potential of the naso-oropharyngeal microbiota compared with local controls, the microbial communities in the two hospitalized patient groups did not differ significantly from each other, indicating a limited impact of SARS-CoV-2 on the naso-oropharyngeal microbiota in hospitalized patients. Besides, we identified *Alloprevotella* and *Solobacterium* as bacterial genera uniquely enriched in COVID-19 patients, which may serve as more specific biomarkers for COVID-19 detection.

## INTRODUCTION

Coronavirus disease 2019 (COVID-19) caused by a novel betacoronavirus, severe acute respiratory syndrome coronavirus 2 (SARS-CoV-2), remains a global public health threat since its emergence at the end of 2019. As of May 2022, over 520 million people were infected worldwide, causing more than six million deaths (https://covid19.who.int/). The clinical symptoms of SARS-CoV-2-infected individuals vary, around 33% of whom exhibit no symptoms at all (1). It was estimated that around 5% of COVID-19 patients develop severe to critical disease (2), with an estimated median infection fatality rate of 0.31% (3). COVID-19 is a systemic disease with multiorgan involvements (4); however, the respiratory tract remains the main organ involved. Respiratory symptoms predominate in symptomatic individuals, with cough (50%), dyspnea (29%), and sore throat (20%) being the main symptoms observed (2).

The upper respiratory tract is the major site of entry for SARS-CoV-2 infections. After inhalation of SARS-CoV-2 laden particles, the viruses can enter human cells through angiotensin-converting enzyme 2 receptors (5, 6). SARS-CoV-2 activates these receptors, inducing inflammation and causing an imbalance, or dysbiosis, of the local and gut microbiota (7). The human upper respiratory tract contains a community of microorganisms comprising the airway and naso-oropharyngeal microbiota, which serves as an essential component of the epithelial barrier and plays a key role in resistance to infection (8, 9). The human respiratory microbiota is important for developing and maintaining immune homeostasis. Previous studies have reported that respiratory microbes can influence the outcome of infectious diseases by regulating the host mucosal immunity (10 to 12). Notably, the bacterial airway microbiota can directly impact influenza virus infection (13, 14) or act indirectly through the host immune system (15, 16).

Previous studies have shown that alterations in the microbiota can affect the onset and progression of infectious diseases, including those caused by influenza A/H7N9 (17), human immunodeficiency virus (18), and hepatitis B virus (19). During viral infection, the balance of the airway microbiota is disrupted to promote host innate immune response, and bacterial colonization may be associated with this process (20, 21). Recent studies utilizing 16S rRNA gene-sequencing analysis have revealed alterations in the nasal and oropharyngeal microbiota of SARS-CoV-2-infected patients compared with normal controls (22 to 25). However, only a few small-scale studies have also included patient controls without SARS-CoV-2 infection for comparison (26 to 29). The inclusion of a disease control group is important because it allows us to differentiate whether the microbiota dysbiosis is caused by SARS-CoV-2 or respiratory diseases in general.

Our group has previously reported that an alteration of the gut microbiota in COVID-19 patients was associated with disease severity and the fecal level of SARS-CoV-2 (30). In this study, we characterized and compared the naso-oropharyngeal microbiome among hospitalized COVID-19 patients (*n* = 76), hospitalized non-COVID-19 patients with respiratory symptoms but tested negative for SARS-CoV-2 infection (*n* = 69), and community controls (*n* = 76) to investigate the impact of SARS-CoV-2 infection on the human microbiota in the upper respiratory tract.

## RESULTS

### Study subjects.

A total of 221 subjects, including 76 hospitalized COVID patients, 69 hospitalized non-COVID patients, and 76 local controls, were enrolled in this study (Table 1; Data Set S1 in the supplemental material). The local controls contained a higher proportion of females than the patient groups, whereas the non-COVID patients were older than the COVID patients and local controls. Pooled nasopharyngeal and throat swab samples from the non-COVID patients and local controls were collected on the same day of recruitment, and the collection day from illness onset of the COVID patients ranged between 0 and 50 days (median = 11 days). The clinical characteristics of COVID patients were classified as previously described (31). Half of the COVID patients were asymptomatic (*n* = 3) or had mild disease without pneumonia (*n* = 35), whereas 32 and 6 of them had moderate (pneumonia) and severe (pneumonia requiring oxygen supplementation)/critical (pneumonia requiring mechanical ventilation) disease, respectively. The non-COVID patients were tested for SARS-CoV-2 due to the presence of fever, respiratory symptoms, shortness of breath, or had an epidemiological link to COVID-19 exposure but subsequently tested negative. Only three of them were admitted for infectious disease, with one laboratory-confirmed Pseudomonas aeruginosa pneumonia and one norovirus gastroenteritis, and one had upper respiratory tract symptoms of sneezing and running nose without microbiological diagnosis. The remaining 66 patients were admitted for noninfectious causes with discharge diagnosis including acute pulmonary edema and congestive heart failure (*n* = 14), renal failure with fluid overload (*n* = 13), asthma and chronic obstructive pulmonary disease exacerbation (*n* = 6), symptomatic anemia (*n* = 3), dementia-related symptoms such as confusion (*n* = 3), gouty arthritis (*n* = 3), cardiovascular accident (*n* = 2), and other miscellaneous noninfectious diagnosis (*n* = 22). All hospitalized patients in our cohort, including COVID-19 and non-COVID-19 groups, survived the admission episode.

**TABLE 1:** Characteristics of study subjects^*a*^

Characteristic	COVID (*n* = 76)	Non-COVID (*n* = 69)	Control (*n* = 76)
Age, Yrs			
Median (range)	35 (2–70)	73 (20–100)	30 (18–63)
≤40	50 (65.8)	6 (8.7)	57 (75)
>40	26 (34.2)	63 (91.3)	19 (25)
Sex			
Male	41 (53.9)	36 (52.2)	22 (28.9)
Female	35 (46.1)	33 (47.8)	54 (71.1)
COVID status	Positive	Negative	Negative
Pneumonia			
Yes	38 (50)	69 (100)	0 (0)
No	38 (50)	0 (0)	76 (100)
Disease severity			
Asymptomatic	3 (3.9)		
Mild	35 (46.1)		
Moderate	32 (42.1)		
Severe/critical	6 (7.9)		
Viral load, Log copies/mL			
≤7.5	33 (43.4)		
>7.5	35 (46.1)		
NA	8 (10.5)		
C-reactive protein, mg/dL			
≤0.5	24 (31.6)		
>0.5	27 (35.5)		
NA	25 (32.9)		

a All values are count (%) unless otherwise specified. NA, not available.

### Hospitalized COVID and non-COVID patients differed in naso-oropharyngeal microbiota compared with local controls.

**FIG 1:**
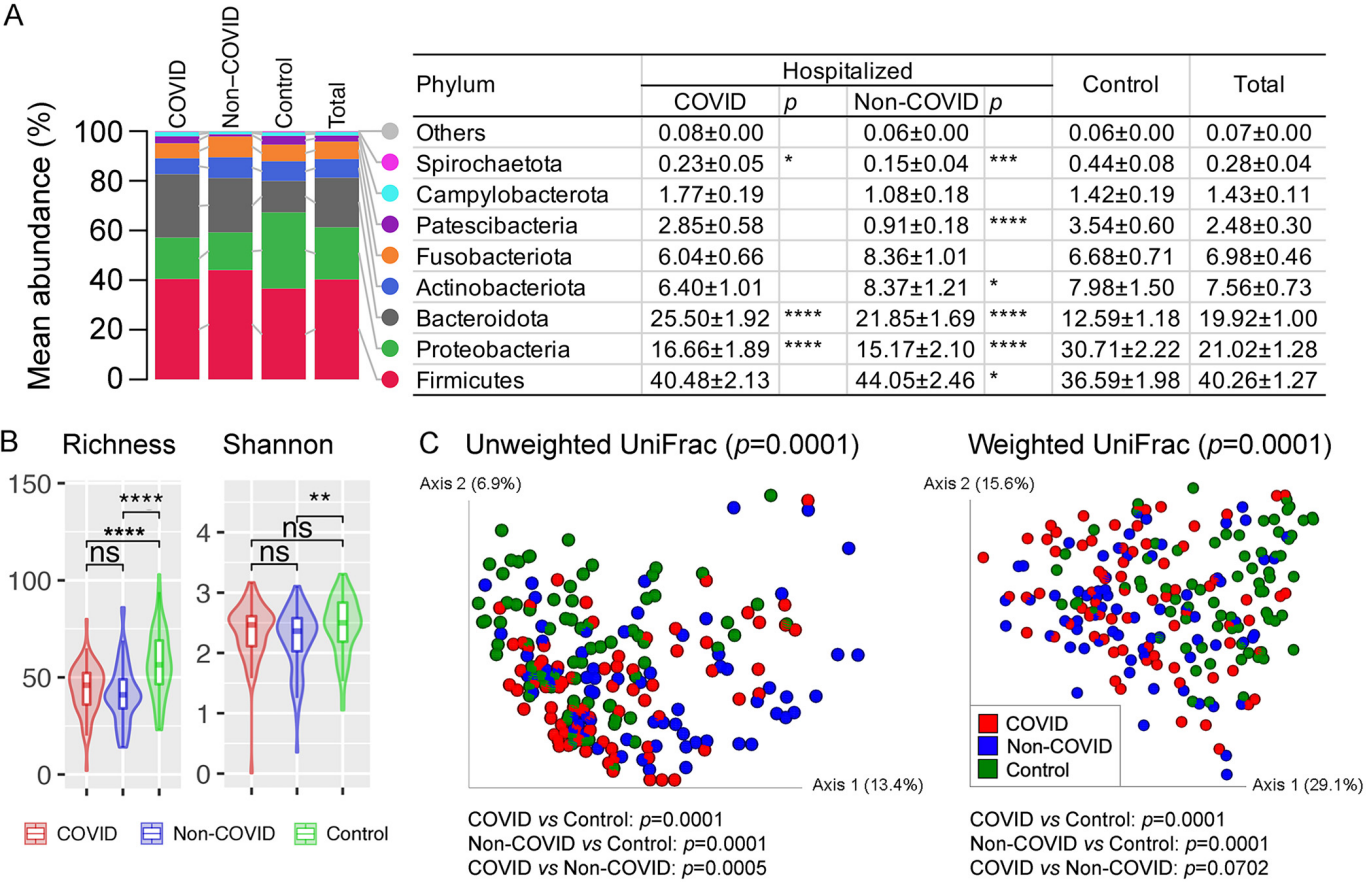
Characterization of the naso-oropharyngeal microbiota in COVID and non-COVID patients and local controls. (A) Relative abundance of the microbiota summarized at the phylum level. Values in the table are mean relative abundance ± standard error of the mean. Phyla with a mean total relative abundance of <0.1% are grouped as Others (Synergistota, Desulfobacterota, Acidobacteriota, Cyanobacteria, Myxococcota, and Bdellovibrionota). Wilcoxon rank-sum test for the difference in relative abundance between hospitalized COVID and control, and between hospitalized non-COVID and control, was performed. (B) Comparison of the naso-oropharyngeal microbiota alpha diversity summarized at the genus level. Pairwise differences between groups were performed using Wilcoxon rank-sum test. (C) Principal coordinates analysis plots based on unweighted and weighted UniFrac distances inferred from amplicon sequence variants (ASVs). *, *P < *0.05; **, *P < *0.01; ***, *P < *0.001; ****, *P < *0.0001; ns, not significant.

A total of 4,910,930 high-quality 16S rRNA V3-V4 reads were generated in this study, ranging between 2,042 and 61,128 reads per sample (22,221 ± 12,726). Firmicutes (relative abundance of 40.3 ± 1.3%) was the predominant bacterial phylum in the naso-oropharyngeal swab samples, followed by Proteobacteria (21.0 ± 1.3%), Bacteroidota (19.9 ± 1.0%), Actinobacteriota (7.6 ± 0.7%), Fusobacteriota (7.0 ± 0.5%), and nine other phyla (Fig. 1A; Fig. S1). Difference in the relative abundance of bacterial phyla, such as significant enrichment of Bacteroidota and reduction of Proteobacteria (Mann Whitney U test: *P ≤ *0.0001), was observed in both hospitalized COVID and non-COVID patients compared with the local controls. Similarly, depressed richness (*P ≤ *0.0001) and/or Shannon diversity (*P ≤ *0.01) of the microbial community at the genus level was observed in COVID and non-COVID patients (Fig. 1B). There was no significant difference of alpha diversity between the COVID and non-COVID patients. Principal coordinates analysis inferred from both unweighted and weighted UniFrac distances of amplicon sequence variants (ASVs) revealed distinct clustering between COVID patients and local controls (*P ≤ *0.0001) and between non-COVID patients and local controls (*P ≤ *0.0001) (Fig. 1C); however, the separation was not significant between hospitalized COVID and non-COVID patients based on weighted UniFrac distance (*P > *0.05).

### Limited number of uniquely enriched bacterial genera in naso-oropharyngeal swabs of hospitalized COVID patients.

**FIG 2:**
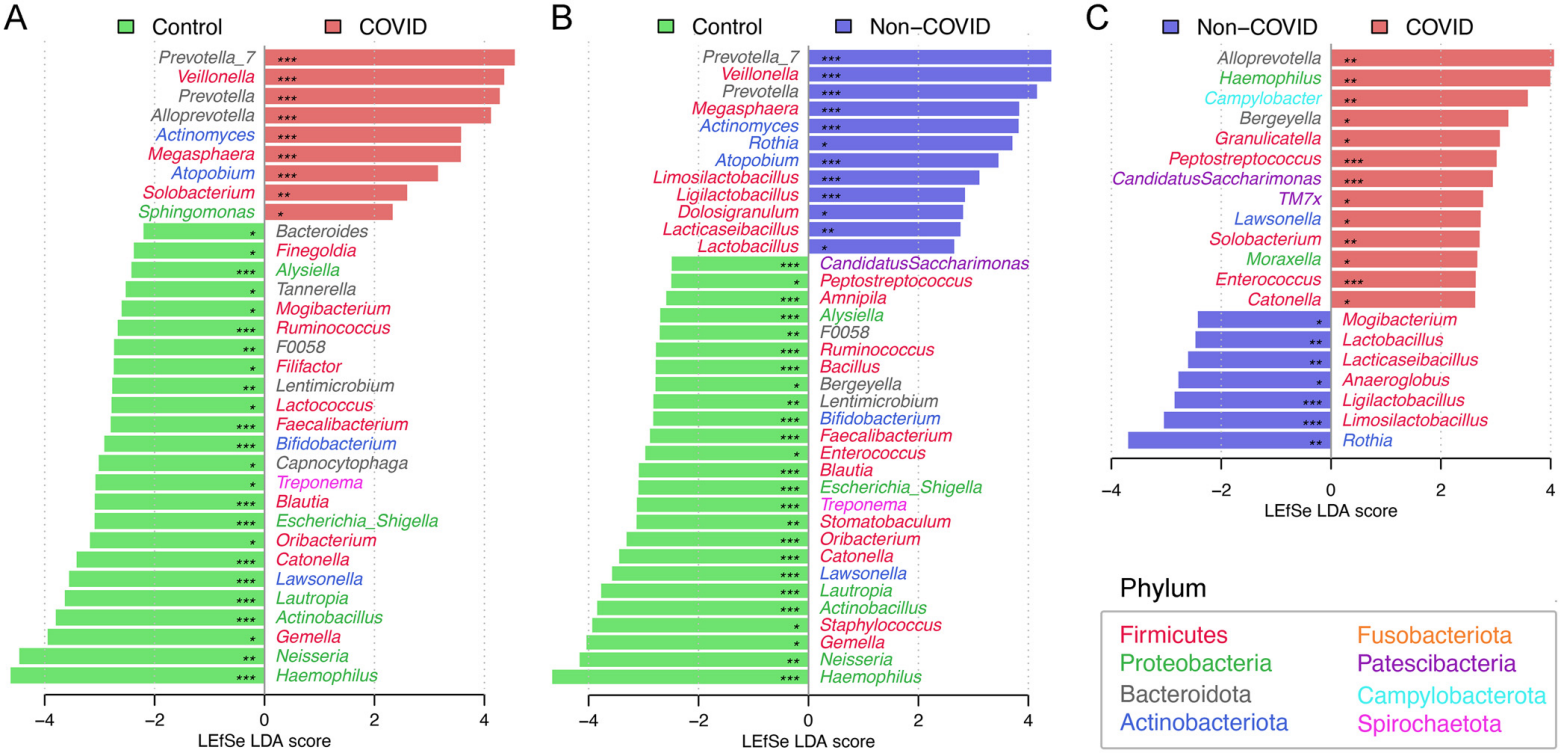
Linear discriminant analysis (LDA) effect size (LEfSe) identifying discriminative bacterial genera (A) between hospitalized COVID patients and local controls, (B) between hospitalized non-COVID patients and local controls, and (C) between hospitalized COVID and non-COVID patients. *, *q *< 0.05; **, *q *< 0.01; ***, *q* < 0.001.

**FIG 3:**
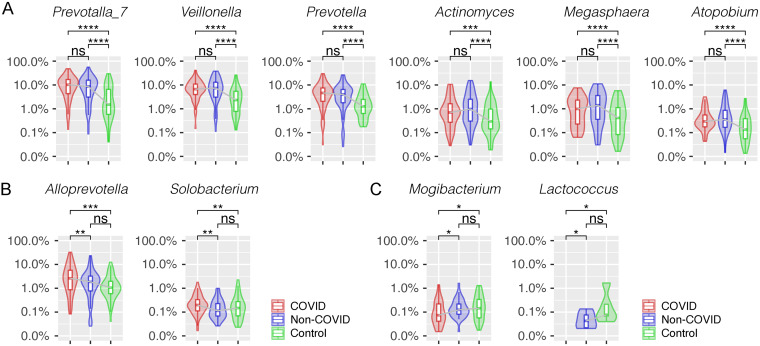
Comparison of the relative abundance of discriminative bacterial genera of COVID patients. (A) Bacterial genera significantly enriched in both COVID and non-COVID patients compared with local controls. Bacterial genera uniquely enriched (B) and depressed (C) in COVID patients compared with non-COVID patients or local controls. *, *P < *0.5; **, *P < *0.01; ***, *P < *0.001; ****, *P < *0.0001; ns, not significant.

We then applied a linear discriminant analysis effect size (LEfSe) test to detect discriminative bacteria in the COVID patients compared with local controls and found nine bacterial genera significantly enriched in the COVID group (*Prevotalla_7*, *Veillonella*, *Prevotella*, *Alloprevotella*, *Actinomyces*, *Megasphaera*, *Atopobium*, *Solobacterium*, and *Sphingomonas*) (Fig. 2A and Data Set S2). Interestingly, six of them were also enriched in the hospitalized non-COVID patients compared with local controls (Fig. 2B and 3A). For example, *Prevotella_7* was enriched in both hospitalized COVID (mean relative abundance of 11.6% versus 4.5%, Mann-Whitney U test: *P ≤ *0.001) and non-COVID patients (10.0% versus 4.5%, *P ≤ *0.001) compared with local controls, but it was not significantly different in relative abundance between the hospitalized COVID and non-COVID patients (*P = *0.223). We also observed suppression of 18 bacterial genera in both hospitalized COVID and non-COVID patients compared with local controls (Fig. S2). Although numerous bacteria distinguishing COVID from non-COVID patients were observed by LEfSe test (Fig. 2C), only two taxa (*Alloprevotella* and *Solobacterium*) were uniquely enriched in the COVID patients (Fig. 3B). In contrast, *Mogibacterium* and *Lactococcus* were dramatically decreased in the COVID patients only, suggesting they may be important markers in differentiating COVID patients from non-COVID patients (Fig. 3C). In addition, *Bifidobacterium* showed a trend of significant reduction in relative abundance from local controls → hospitalized non-COVID patients → COVID patients (0.17% versus 0.03% versus 0.002%, *P ≤ *0.001).

### Hospitalized COVID and non-COVID patients shared a similar naso-oropharyngeal microbiota.

**FIG 4:**
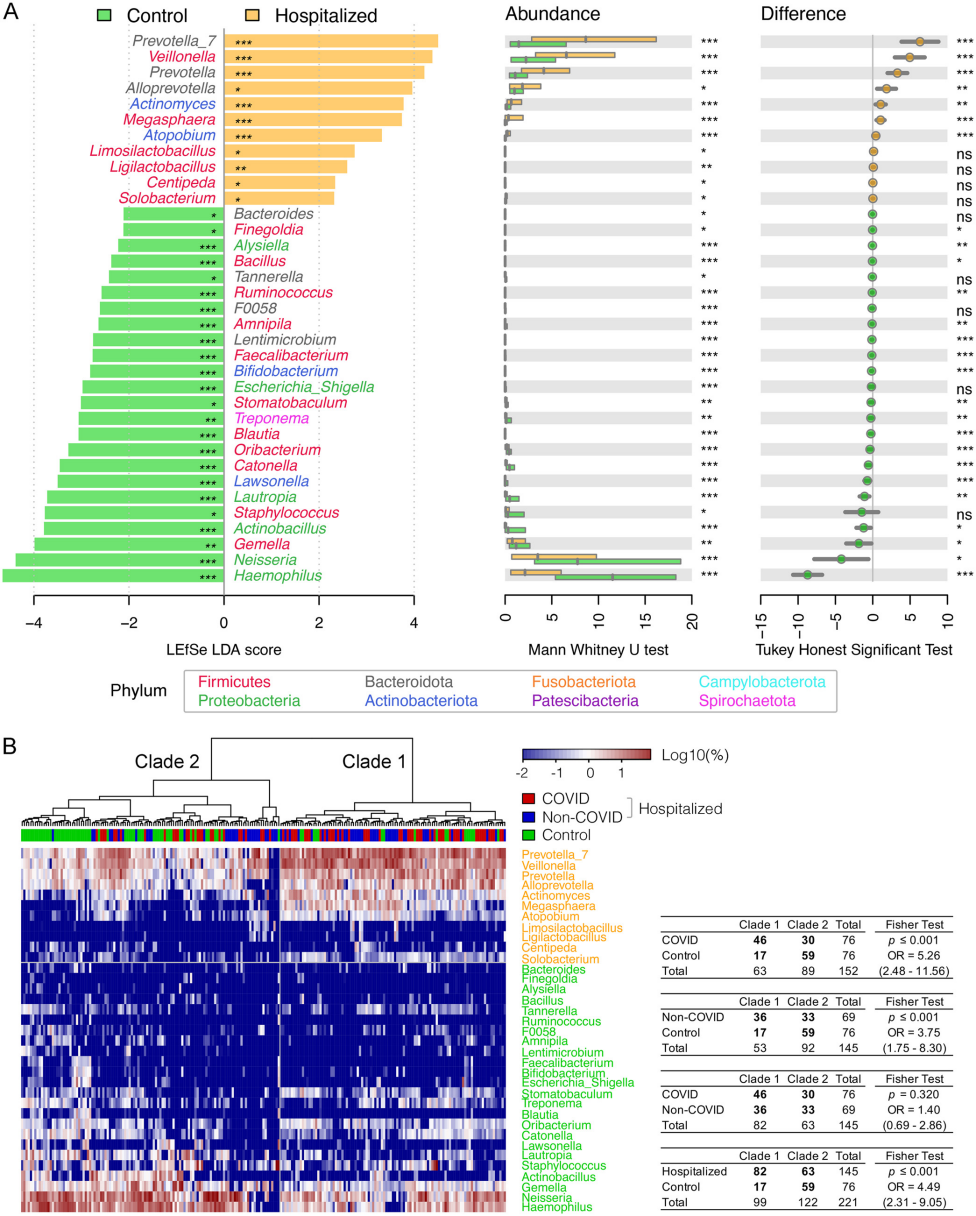
Dysbiosis of the oropharyngeal microbiota in hospitalized patients (COVID and non-COVID) compared to local controls. (A) Discriminative bacterial genera distinguishing hospitalized patients from local controls by LEfSe. Difference in the relative abundance tested by pairwise Mann-Whitney U test and Tukey’s HSD *post hoc* test are shown on the right panels. (B) Hierarchical cluster analyses using distance matrix of discriminative bacterial genera classified the surveyed samples into two clades based on the dendrogram topologies, with a heat map indicating the relative abundance.

As the naso-oropharyngeal microbiota was similar in alpha and beta diversity between hospitalized COVID and non-COVID patients, we then used LEfSe to characterize bacteria with differential abundance in the overall hospitalized patients (COVID and non-COVID) compared to local controls (Fig. 4A). A total of 11 and 24 enriched and depressed bacterial genera, including the six taxa enriched in both COVID and non-COVID patient groups, were identified, respectively; among them, 10 discriminative bacteria had area under the receiver operating characteristic curve (AUC) values of ≥0.70 (a combined AUC of 0.92, 95% CI 0.88 to 0.97) to distinguish hospitalized patients from local controls, highlighting a potential utility of bacterial markers to manage hospitalized patients with respiratory tract symptoms (Fig. S3). Although a hierarchal cluster analysis using these discriminative bacteria well divided the surveyed samples into two clades, mainly between the hospitalized patients and local controls (*P ≤ *0.001), the separation between COVID and non-COVID groups was not significant (*P = *0.320) (Fig. 4B), which confirms that hospitalized COVID and non-COVID patients shared a similar naso-oropharyngeal microbiota.

### Hospitalized COVID and non-COVID patients shared a similar predicted naso-oropharyngeal metabolism.

**FIG 5:**
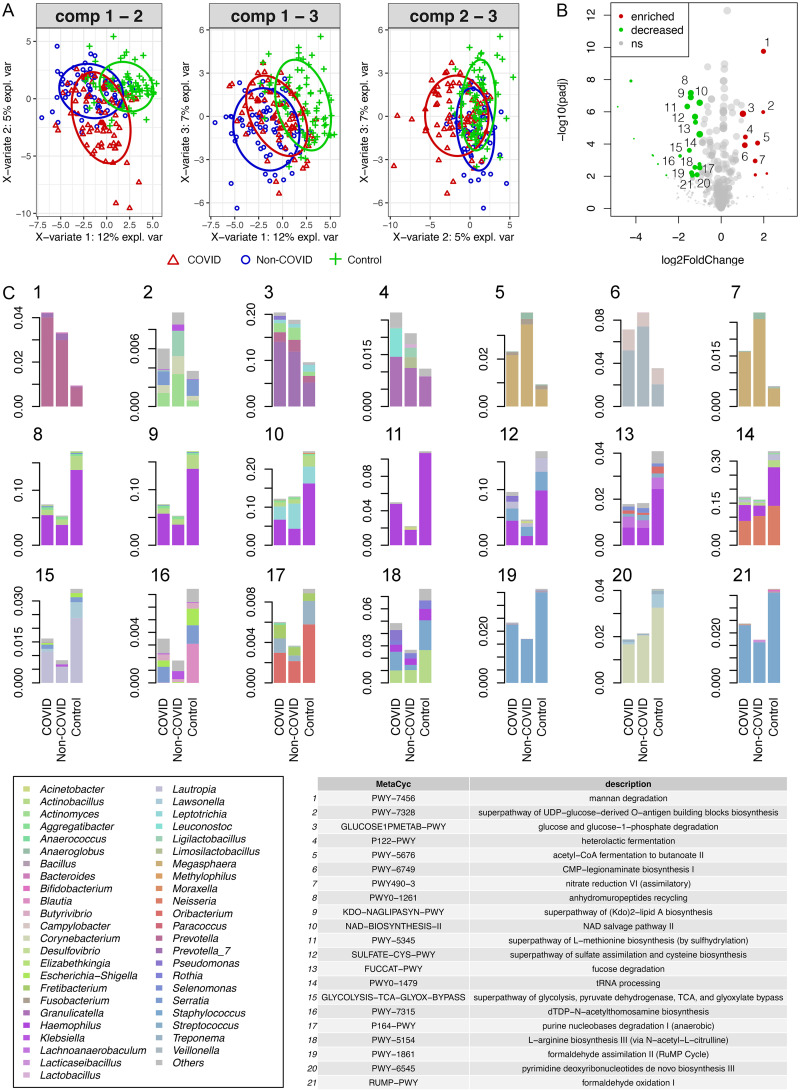
Comparison of metabolic pathways enriched in hospitalized COVID and non-COVID patients compared with local controls. (A) Principal component ordination of variance stabilization-transformed MetaCyc pathway abundances predicted by PICRUSt2. (B) Volcano plot showing differentially dysregulated pathways between hospitalized patients (COVID and non-COVID) and local controls identified using binomial generalized log-linear model in EdgeR (abs(log2FC) > 1, *q *≤ 0.01, baseMean > 50). Dots in red and green indicate enriched and decreased pathways, respectively. (C) Bar plots showing differentially dysregulated pathways between hospitalized COVID patients, non-COVID patients, and local controls.

Dysbiosis in the naso-oropharyngeal microbiota in the COVID patients may alter the metabolic functions of the microbiota. To test this hypothesis, we used PICRUSt2 to predict metabolic profiles based on the 16S rRNA sequences. Sparse partial least-squares discriminant analysis (sPLS-DA) performed on variance stabilized-transformed MetaCyc pathway profiles revealed discriminative clustering between COVID patients and local controls, and between non-COVID patients and local controls (Fig. 5A). However, COVID and non-COVID patients tended to have functionally similar communities. Among those differentially abundant metabolic pathways (baseMean ≥ 50, abs(log2FC) ≥ 1, *p_adj_* ≤ 0.01) (Fig. 5B), small differences were observed between the hospitalized COVID and non-COVID patients (Fig. 5C and Data Set S3). Seven metabolic pathways were enriched in the hospitalized patients, including heterolactic fermentation and nitrate reduction VI (assimilatory). In contrast, 21 metabolic pathways were enriched in the local controls, including anhydromuropeptides recycling and NAD salvage pathway II.

### Limited association between demographic and clinical parameters and naso-oropharyngeal microbiota in COVID-19 patients.

To explore possible relationships between the naso-oropharyngeal microbiota and demographic and clinical parameters in COVID patients, we first associated the alpha and beta diversity of the microbiota with sex, age, pneumonia status, C-reactive protein (CRP) level, and peak viral load of the COVID patients. No significant difference in the alpha or beta diversity of the microbial community was detected among groups in most of these variables, except for age, which was significantly associated with the bacterial richness and beta diversity in COVID patients (*P ≤ *0.05) (Fig. S4 and S5).

**FIG 6:**
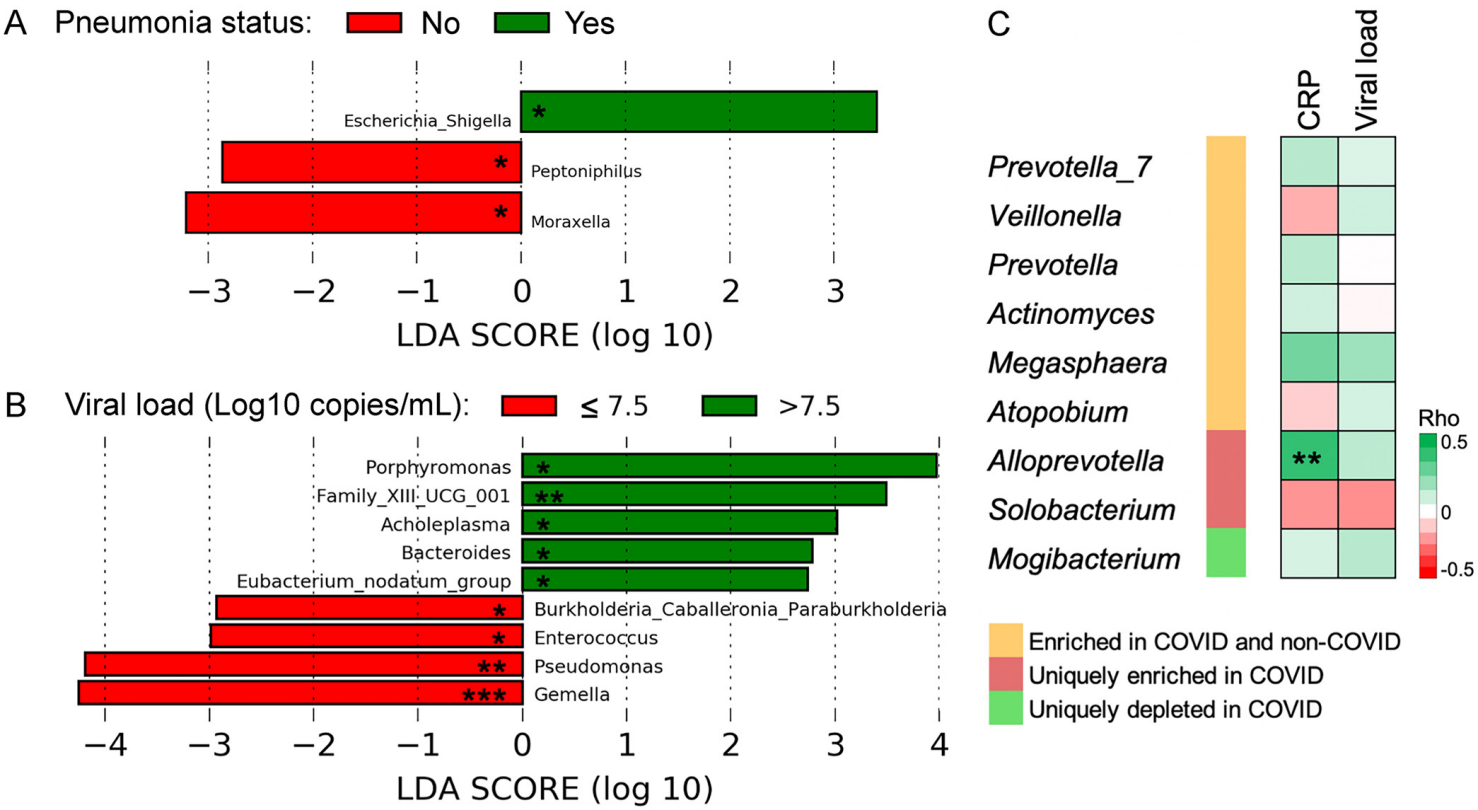
Associations between clinical parameters and the relative abundance of the naso-oropharyngeal microbiota in COVID patients. Discriminative bacterial genera between COVID patients with different (A) pneumonia status and (B) viral load (Log_10_ copies/mL). *, *P < *0.05; **, *P < *0.01; ***, *P < *0.001. No discriminative bacterial genera were detected between COVID patients with different CRP levels. (C) Spearman’s correlations between discriminative bacterial genera of COVID patients and clinical variables. CRP, C-reactive protein. **, *P < *0.01. Correlations for *Lactococcus* were not available because this genus was not detected in COVID patients.

We then used LEfSe to identify discriminative bacterial genera associated with the clinical parameters. Results showed that *Escherichia_Shigella* was enriched in COVID patients with pneumonia (Fig. 6A) and five genera, including *Porphyromonas*, *Family XIII UCG 001*, and *Acholeplasma*, were enriched in COVID patients with a high peak viral load (>7.5 Log_10_ copies/mL) (Fig. 6B). No discriminative bacterial genera were detected between COVID patients with different CRP levels. Besides, we examined the correlations between the discriminative bacterial genera of COVID patients and clinical variables. Results revealed a significant positive correlation between the relative abundance of *Alloprevotella*, one of the uniquely enriched genera in COVID patients, and CRP level (Spearman's Rho = 0.4, *P* < 0.01) (Fig. 6C). In contrast, none of the discriminative bacterial genera of COVID patients were significantly correlated with the peak viral load.

## DISCUSSION

While an altered naso- or oro-pharyngeal microbiota in COVID-19 patients compared with normal controls is well received (22 to 25), contrasting results have been reported on whether the naso- or oropharyngeal microbiota differs between COVID-19 and non-COVID-19 patients (27, 28, 32, 33). These studies typically have a small sample size for the disease control group (*n* < 30). As a result, any resemblance detected may be due to the lack of statistical power instead of genuine similarity between the two groups. In this study, we characterized and compared the naso-oropharyngeal microbiota among 76 hospitalized COVID-19 patients, 69 hospitalized non-COVID-19 patients, and 76 local community controls and observed no significant difference in the alpha and beta diversity between the microbiota of COVID and non-COVID patients. As these non-COVID patients had respiratory symptoms or related illnesses similar to that of the COVID-19 patients, our results suggest that while respiratory disease outcomes are associated with dysbiosis of the naso-oropharyngeal microbiota, the direct impact of SARS-CoV-2 is probably limited.

*Prevotella_7*, *Veillonella*, and *Prevotella* were among the top bacterial genera enriched in the naso-oropharyngeal microbiota of COVID patients compared with local controls. This is in agreement with previous studies, which revealed the enrichment of *Prevotella* and *Veillonella* in the oropharyngeal microbiota of COVID-19 patients (24, 34). In fact, members from both genera exhibit proinflammatory properties, and their relative abundances in the oral microbiota were found to be associated with long COVID (35). Notably, the relative abundance of *Prevotella* in the nasopharyngeal microbiota was found to be positively associated with COVID-19 severity (36), which may be due to its association with the nasopharyngeal cytokines CCL2 and VEGF (23). Previous studies have shown that both *Prevotella* and *Veillonella* can facilitate the progression of respiratory diseases, especially those associated with mucus accumulation, by degrading mucin molecules on the respiratory mucosa, thereby enabling the otherwise impossible colonization and growth of pathogenic bacteria (37, 38). Specifically, enriched metabolic pathways involved in mannan degradation (PWY-7456), glucose and glucose-1-phosphate degradation (GLUCOSE1PMETAB-PWY), and CMP-legionaminate biosynthesis I (PWY-6749) in the surveyed hospitalized patients were mainly contributed by the enrichment of *Prevotella* and *Veillonella*. Therefore, while these two bacterial genera may facilitate the progression of COVID-19 by promoting secondary infections in the patients, they are probably not unique in COVID-19 nor specifically involved in SARS-CoV-2 infection.

To identify bacterial taxa in the naso-oropharyngeal microbiota that are uniquely linked to SARS-CoV-2 infection, we compared the results of differential abundance analysis across groups and revealed that *Alloprevotella* and *Solobacterium* were the only bacterial genera uniquely enriched in COVID-19 patients. *Alloprevotella* is a genus of obligately anaerobic, nonmotile, Gram-negative bacilli closely related to the genus *Prevotella*. *Alloprevotella* was identified to be an indicator of one of the COVID-19-related community types of the oropharyngeal microbiota (29), and its relative abundance in the nasopharyngeal microbiota was found to be positively associated with COVID-19 severity (36). Besides, *Alloprevotella* was found in the oral microbiota of patients with oral squamous cell carcinoma and halitosis (39, 40). *Solobacterium* is a genus of obligate anaerobic non-spore-forming Gram-positive bacilli and one of the predominant bacterial genera detected in the oropharyngeal microbiota of COVID-19 patients (27). *S. moorei*, the only species in the genus, played a role in halitosis when enriched in the oral cavity (41). Since these two genera of opportunistic pathogens were specifically enriched in the naso-oropharyngeal microbiota of COVID-19 but not in non-COVID-19 patients, they may represent more specific biomarkers for detecting SARS-CoV-2 infection. In contrast, *Mogibacterium*, *Lactococcus*, and *Bifidobacterium* were significantly depleted in the surveyed COVID-19 patients compared with non-COVID patients and/or the local controls. Much lower level of *Mogibacterium* has been reported in COVID-19 patients requiring ICU (42); several species within the genera *Lactococcus* and *Bifidobacterium* play important roles in modulating innate cytokine and immune response, which potentially provides a protective benefit against COVID-19 if the naso-oropharyngeal microbiota approximating healthy condition is restored (43).

We predicted the metabolic functions of the naso-oropharyngeal microbiota using PICRUSt2. Results revealed the enrichment of seven metabolic pathways in the hospitalized (COVID and non-COVID) patients, including heterolactic fermentation and nitrate reduction VI (assimilatory). Heterolactic fermentation is one of the pathways enriched in respiratory samples from hospitalized cystic fibrosis patients at pulmonary exacerbation onset compared to those collected at the end of antibiotic treatment (44). Besides, nitrate reduction VI (assimilatory) is enriched in the gut microbiota of patients with unruptured intracranial aneurysm, a condition driven by chronic inflammation, compared to healthy controls (45).

We performed association analysis to examine if there is any association between the naso-oropharyngeal microbiota of COVID-19 patients and demographic and clinical variables. No significant association was detected between the alpha or beta diversity of the microbiota and the pneumonia status (a proxy of disease severity), CRP level (a marker for inflammation), or viral load. This is contrary to other studies, which reported a lower alpha diversity in the oropharyngeal microbiota of critically ill COVID-19 patients (27). The discrepancy may be due to the relatively few critically ill patients recruited in our cohort. We found that age was significantly associated with the alpha and beta diversity of the naso-oropharyngeal microbiota of COVID-19 patients. This is in concordance with previous findings that distinct age groups of COVID-19 patients harbored distinct microbial groups in the nasopharyngeal microbiota (46). In fact, age is among one of the host factors that may impact COVID-19 disease severity via interactions with the oropharyngeal microbiota (27). Importantly, we revealed a positive correlation between the relative abundance of *Alloprevotella* in the naso-oropharyngeal microbiota and the CRP level of COVID-19 patients. This finding is in good agreement with the positive association previously reported between *Alloprevotella* in the nasopharyngeal microbiota and COVID-19 severity (36), indicating an important role of *Alloprevotella* in the pathogenesis of COVID-19.

The main strengths of our study are the relatively large number of subjects recruited across the three comparison groups, and the inclusion of both hospitalized patient controls and local community controls. The inclusion of two control groups allows us to reveal a limited impact of SARS-CoV-2 on the naso-oropharyngeal microbiota in hospitalized patients despite significant alterations, compared with normal controls. Limitations of our study include a small number of asymptomatic and severe/critical COVID-19 patients, which has hindered detailed association analysis between the naso-oropharyngeal microbiota and disease severity. The different age distributions among groups could have also influenced the microbiome results. Besides, the lack of follow-up samples collected from the same COVID-19 patients has precluded longitudinal analysis. Lastly, the use of 16S rRNA amplicon sequencing instead of shotgun metagenomic sequencing has hampered detailed investigations into the virulence factors and pathways associated with COVID-19 pathogenesis.

In conclusion, our study has revealed an alteration of the naso-oropharyngeal microbiota in COVID-19 patients compared with local community controls, which resembled that in non-COVID-19 patients with similar respiratory symptoms, highlighting a limited impact of SARS-CoV-2 on the naso-oropharyngeal microbiota. *Alloprevotella* and *Solobacterium* were uniquely enriched in the naso-oropharyngeal microbiota of COVID-19 patients, suggesting an important role of these bacteria in COVID-19 pathogenesis. Moreover, these bacteria may represent more specific biomarkers for COVID-19 detection.

## MATERIALS AND METHODS

### Ethics approval.

All subjects provided written consent to participate. This study was approved by The Joint Chinese University of Hong Kong—New Territories East Cluster Clinical Research Ethics Committee.

### Subject recruitment.

Three subject cohorts were recruited between April and October 2020, including (i) hospitalized COVID-19 patients admitted to the Prince of Wales Hospital who tested positive for SARS-CoV-2 by RT-PCR targeting the nucleocapsid (N) gene (here referred to as the COVID group); (ii) hospitalized patients admitted to the same hospital during the same period for the presence of respiratory symptoms or related illnesses but tested negative for SARS-CoV-2 (non-COVID); and (iii) volunteer subjects recruited from the local community without known disease or any upper respiratory symptoms and tested negative for SARS-CoV-2 (local control). None of the subjects received antimicrobial therapy within 2 weeks prior to sample collection.

### Sample processing and RT-PCR for SARS-CoV-2.

Pooled nasopharyngeal and throat swabs were collected from each subject in a sterile bottle with viral transport medium (Remel MicroTest M4RT, Thermo Fisher) as previously described (47). Both DNA and RNA were extracted using the QIAamp Viral RNA minikit (Qiagen, Germany). SARS-CoV-2 RNA was detected and quantified by RT-PCR targeting the N gene as previously described (48). Samples were considered to be negative if the Ct values exceeded 39.9 cycles. The detection limit of real-time RT-PCR was 694 copies/mL.

### Microbial 16S rRNA gene V3-V4 amplicon sequencing.

The microbial 16S rRNA gene hypervariable V3-V4 region was targeted to characterize the naso-oropharyngeal microbial community using universal primers 341F (5′-CCT ACG GGN GGC WGC AG-3′) and 806R (5′-GGA CTA CNV GGG TWT CTA AT-3′) as previously described (49). Successful amplicons indexed with a pair of dual unique 12-bp barcodes were equally pooled and paired-end sequenced on an Illumina MiSeq (2 × 300 bp). For quality control, a mock community, DNA negative controls, and technical replicates were also sequenced.

### Bioinformatics analysis.

QIIME2 (v2020.11) (50) was used for quality filtering, DADA2 denoising, and taxonomic assignment against the latest SILVA rRNA database (v138) (51). All reads assigned to archaea, mitochondria, and chloroplasts were excluded. Bacterial taxa with ≥1% relative abundance in at least one sample were retained. Here, we used a cutoff 2,000 reads to exclude samples with a low sequencing depth. Alpha diversity indexes, including richness and Shannon diversity, were calculated using the *vegan* package and statistically compared between groups using the Wilcoxon rank sum test in R v3.4.0. Beta diversity among subjects was evaluated using permutational multivariate analysis of variance (PERMANOVA) based on weighted and unweighted UniFrac distances using the beta-group-significance pipeline with 9,999 permutations and visualized in principal coordinates analysis (PCoA) plots using the core-metrics-phylogenetic pipeline in QIIME2. Linear discriminant analysis (LDA) effect size (LEfSe) was used for differential abundance analysis with the default parameters (LDA score > 2, *P ≤ *0.05) (52). A two-sided *P* value or a false discovery rate (FDR)-adjusted *P* value (*q* value) of ≤0.05 was used as the threshold for statistical significance. PICRUSt2 was used to predict bacterial MetaCyc pathways based on the 16S rRNA reads (53).

### Data availability.

All sequence reads generated in this study have been deposited to the NCBI Sequence Read Archive (SRA) under Bioproject accession PRJNA822681.
