# Supplementary material for: Limited Impact of SARS-CoV-2 on the Human Naso-Oropharyngeal Microbiota in Hospitalized Patients
Source: Microbiol Spectr. 2022 Nov 9;10(6):e02196-22. doi: 10.1128/spectrum.02196-22 (PMC9769582; doi:10.1128/spectrum.02196-22)
Supplement: Supplemental file 1 — Fig. S1 to S5. Download spectrum.02196-22-s0001.pdf, PDF file, 2.1 MB [file spectrum.02196-22-s0001.pdf]

Supplemental Figures:

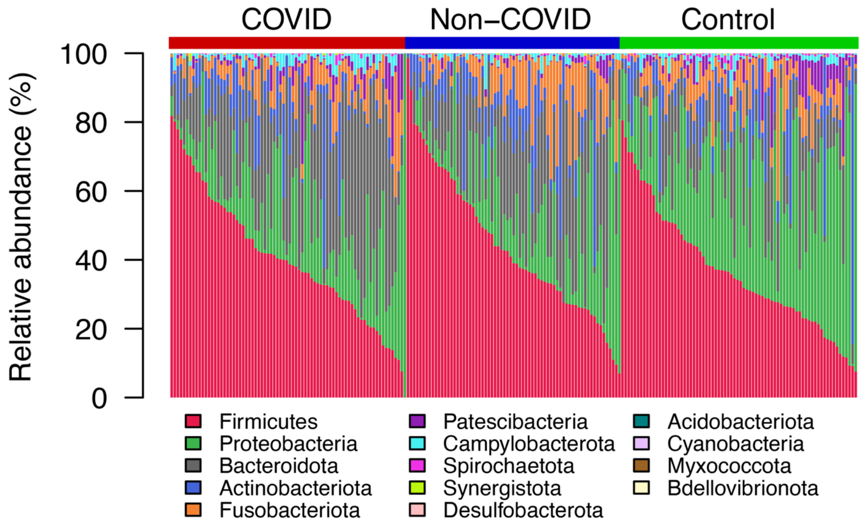

FIG S1 Composition of the naso-oropharyngeal microbiota in each study subject summarized at the phylum level.

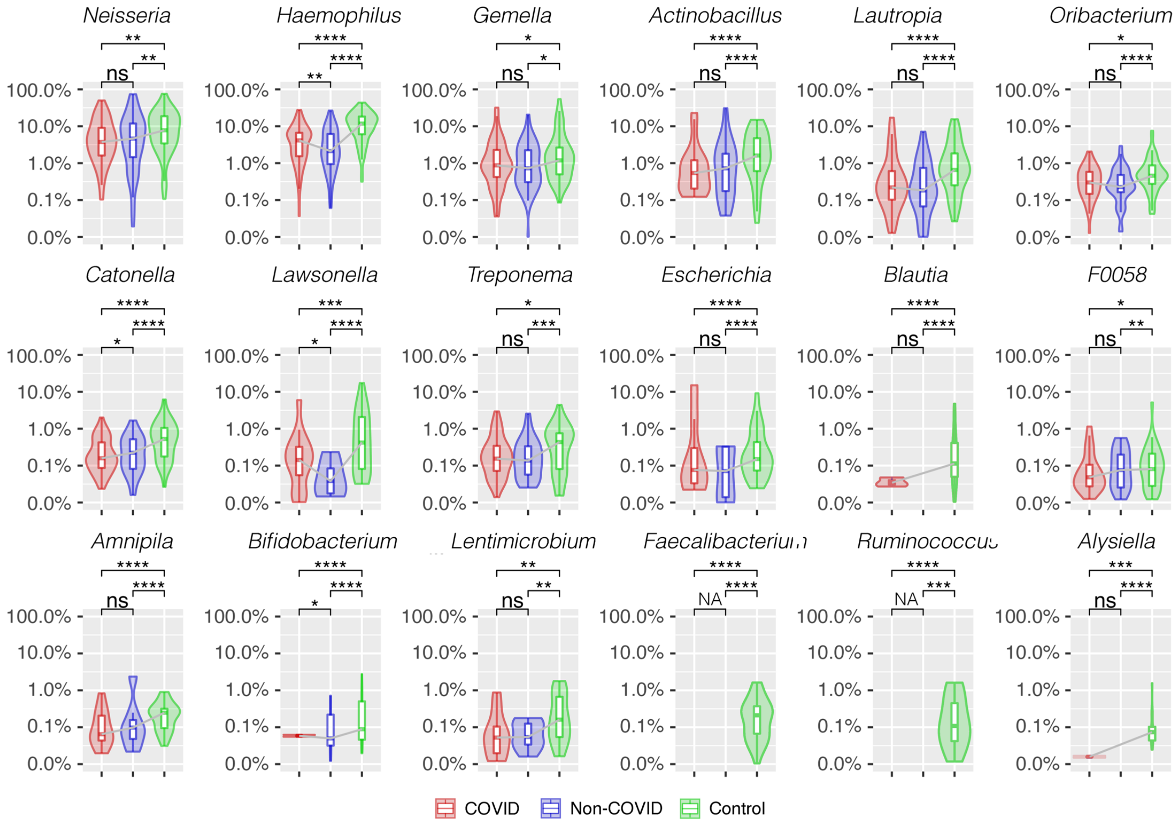

FIG S2 Comparison of the relative abundance of discriminative bacterial genera significantly depressed in both hospitalized COVID and non-COVID patients when compared with local controls. **, *p* < 0.01; ***, *p* < 0.001; ****, *p* < 0.0001; ns, not significant.

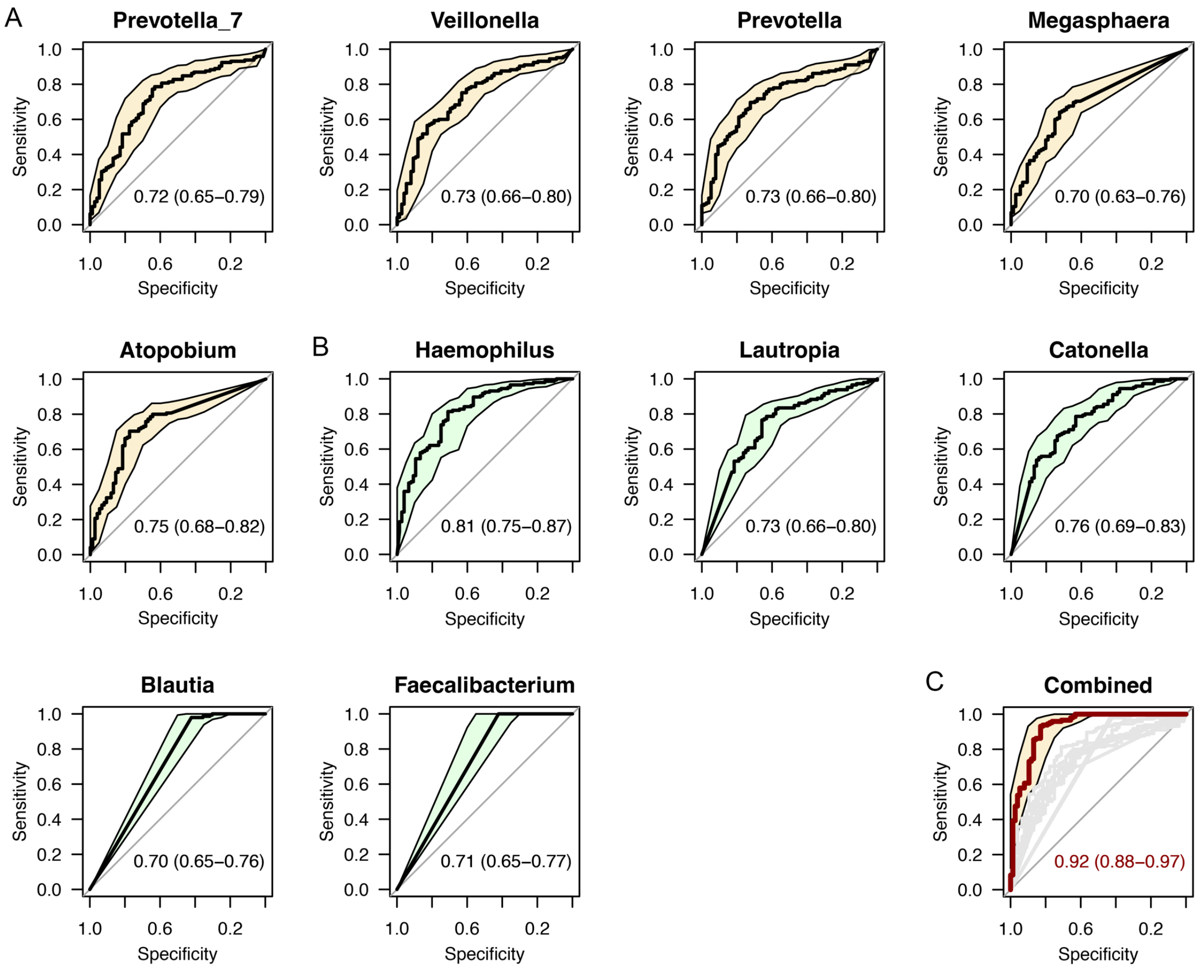

FIG S3 Receiver operating characteristic (ROC) analysis with area under the receiver operating curve (AUC) values distinguishing hospitalized patients (COVID and non-COVID) from local controls. **(A)** Five enriched and **(B)** five depressed bacterial genera with AUC ≥ 0.7 in both COVID and non-COVID patients achieved a combined AUC value of 0.92 **(C)**.

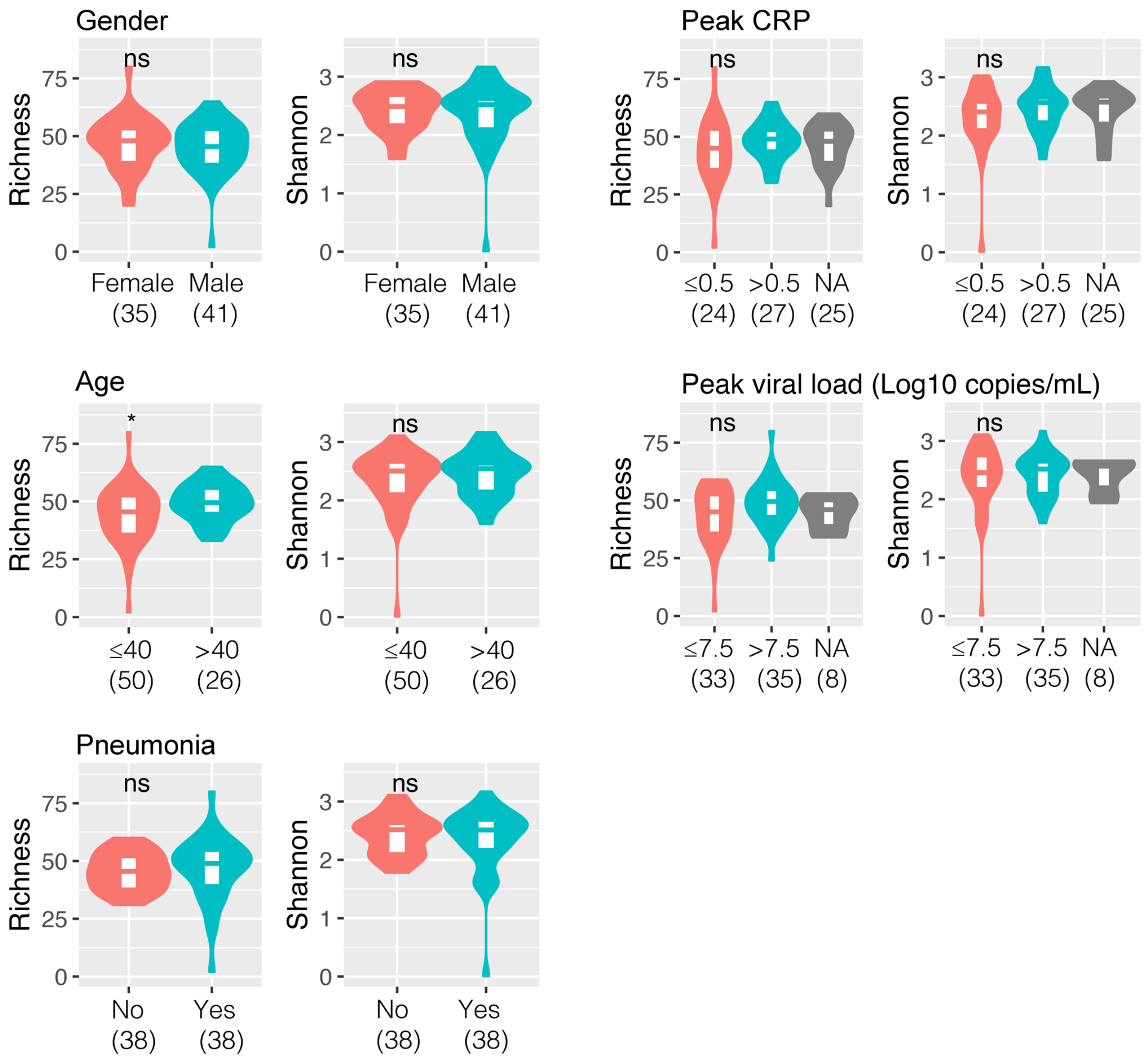

FIG S4 Associations between demographic and clinical parameters and the alpha diversity of the naso-oropharyngeal microbiota in COVID patients. CRP, C-reactive protein; NA, not available. *, $p < 0.05$; ns, not significant.

	Df	SumOfSqs	R2	F	Pr(>F)	
Pneumonia	1	0.1444	0.01133	0.8613	0.613	
Age	1	0.3315	0.02603	1.9779	0.020	*
Sex	1	0.1847	0.01450	1.1019	0.303	
loadSample	2	0.3652	0.02867	1.0893	0.312	
loadPeak	2	0.3477	0.02730	1.0372	0.383	
crp	2	0.3021	0.02372	0.9012	0.609	
Residual	66	11.0631	0.86846			
Total	75	12.7387	1.00000			

FIG S5 PERMANOVA results inferred from weighted UniFrac distance shows no relationship between the beta diversity of the naso-oropharyngeal microbiota in COVID patients and most host variables except age. CRP, C-reactive protein. *, $p < 0.05$.
